# Impact of Family Cohesion and Adaptability on Academic Burnout of Chinese College Students: Serial Mediation of Peer Support and Positive Psychological Capital

**DOI:** 10.3389/fpsyg.2021.767616

**Published:** 2021-12-13

**Authors:** Jincong Yu, Yifan Wang, Xiaoqing Tang, Yuqin Wu, Xuemei Tang, Jie Huang

**Affiliations:** ^1^Education and Counseling Center for Psychological Health, Zhongnan University of Economics and Law, Wuhan, China; ^2^School of Philosophy, Zhongnan University of Economics and Law, Wuhan, China; ^3^School of Foreign Languages, Zhongnan University of Economics and Law, Wuhan, China; ^4^School of Marxism, Wuhan Railway Vocational College of Technology, Wuhan, China

**Keywords:** family environment, academic burnout, peer support, positive psychological capital, serial mediation

## Abstract

This study aimed to explore the association between the functioning of family environment (i.e., family cohesion and family adaptability) and academic burnout of Chinese college students as well as the mediating effects of the interpersonal resource (i.e., peer support) and intrapersonal resource [i.e., positive psychological capital (PsyCap)] in this relationship. A total of 1971 Chinese undergraduates were involved in an online questionnaire survey and data analysis. It was found that family cohesion and adaptability was negatively related to academic burnout. Mediation analyses demonstrated that family cohesion and adaptability did not directly affect academic burnout, but indirectly through increasing PsyCap (characterized by optimism, hope, resilience, and self-efficacy), and through enhancing peer support and then increasing PsyCap in serial. Meanwhile, the relationship between family cohesion and adaptability and academic burnout was not mediated by peer support alone. These findings highlight the family environment’s crucial role in youth mental health and positive development in the college context.

## Introduction

Academic (or learning) burnout is typically defined as a state in which students become emotionally exhausted due to academic demands, cynical toward study and school, and feel inefficacy as a student (Schaufeli et al., 2002; Kiuru et al., 2008). It has been experienced by students of all ages and drawn the attention of the researchers. Generally, burnout is a prolonged response to demands and stressors, usually occurring as a result of depletion of emotional and mental resources needed to provide meaning and value of work of an individual (Salmela-Aro and Upadyaya, 2014). College students, as emerging adults inherently fraught with risks and challenges, usually experience academic, social, and psychological stressors. Among Chinese college students, behavior problems related to burnout symptoms like cyber addiction, playing truant, cheating in exams are quite common, and mild, moderate, and high level of academic burnout were shown among them at 23.5, 43.0, and 7.0%, respectively (Da and Gao, 2015). Moreover, it was reported that 20%–50% of the US medical students screened positive for depression and 30–60% for academic burnout (Lucchetti et al., 2018). The severity of its consequences is even harder to be overlooked. Academic burnout closely relates to poor academic performance (Schaufeli et al., 2002), low commitment to college (Navarro-Abal et al., 2018), negative learning experience (Charkhabi et al., 2013), school dropout (Salmela-Aro et al., 2009), and a host of physical and mental issues such as headaches, anxiety, and depression (Luo et al., 2020). A survey among medical students found that burnout syndromes may become worse by year of schooling and lay at the root of professional burnout and job burnout, so chronic accumulation of burnout syndromes needs to be prevented at the earlier stage (Dyrbye et al., 2006).

What contributes to academic burnout has been explored and empirically evidenced by existing research, including individual factors (personality characteristics such as perfectionism, self-esteem, and self-control) (Chang et al., 2016; Seibert et al., 2016), and school-related factors (demands and pressure in school, instructors’ attitude and behaviors, and peer group influence, etc.) (Walburg, 2014; Chunming et al., 2017; Romano et al., 2021). Family environment is conceptualized as a multi-faceted construct that encompasses a host of factors, such as family structure, parental beliefs, parent-child interactions, and parenting styles as well as cultural and emotional atmosphere. There is a wealth of research evidence indicating that the family environment plays a vital role in shaping personalities, self-concept, and social competence of children (Vondra et al., 1990; Nakao et al., 2000; Luecken et al., 2013; Leto et al., 2019; Xie et al., 2021). The well-being of children will benefit from the family environment of high quality through cognitive, social, behavioral, and emotional pathways because a healthy parent–child relationship can provide necessary support and guidance as protection and buffer against strain and danger (Thomas et al., 2017). Specific factors of family environment, such as family intimacy and conflict, socio-eco status, have been demonstrated to affect academic burnout among middle school students (Luo et al., 2016, 2020). Ma (2021) pointed out that the family environment, which highly relates to intimacy, emotional expression, independence, control, and organization, etc, has a significant effect on academic emotions and the problematic environment of families could affect the self-control of individuals and further induce burnout and depression. Comparatively, little research has explored the impact of family environment on academic burnout in regard to college students. Unlike most of the undergraduates in Western countries, Chinese undergraduates tend to be more economically dependent on their families who are normally responsible for their tuition payments and living expenses. Parents hold extremely high expectations for youth’s academic achievements and employment prospects (Zhang and Carrasquillo, 1995). Moreover, living in a collective society, family members in China are more mentally connected to each other and prone to have a prominent sense of belongings (Nelson et al., 2004). Therefore, the impact of family environment on academic burnout among college students is worth studying.

### Family Cohesion and Adaptability With Academic Burnout

Among factors of family environment, cohesion and adaptability were identified as two basic functions of the family system which are irreplaceable by a peer group or any other social structure across the lifespan (Olson et al., 1979). Family cohesion refers to the emotional bond among family members and the degree of member’s autonomy, while family adaptability is described as the ability to change in response to situational and developmental stress in the family system, such as discipline negotiation style and power structure. In a functional family environment, it is supportive, expressive, and organized, and family members have positive communications, a sense of belongingness, clearly defined rules, and less conflicts. Increasing research have suggested the protective role of family cohesion and adaptability against academic burnout. For instance, a study among 344 middle school students demonstrated that students with less academic burnout showed a higher level of family cohesion and adaptability than those with more burnout (Liao, 2013). Verbal and non-verbal affection and confirmation expressed by parents predict less stress on children by stifling the chance of burnout and improving well-being (Schrodt et al., 2007). Family cohesion and adaptability can negatively affect school burnout through emotional support and enhanced problem-solving skills among high school students (Yin, 2019). When facing adversities in the study, students with higher family cohesion and adaptability could adapt to the environment and figure out the most applicable learning methods to bridge over the troubled water, largely reducing the chance to develop into academic burnout (Hou and Liang, 2017).

### Peer Support and Positive Psychological Capital as the Possible Mediators

The impact of family cohesion and adaptability on academic burnout appears to be crucial, but less is known about the underlying mechanism regarding how a positive and functional family environment protects against academic burnout. According to the job demands-resources (JD-R) theory, job demands including stressful events, heavy workload, and pressure lead to the depletion of energy, while job resources which refer to physical, psychological, social, and organizational aspects of the job, including social support, sense of control, and autonomy, are functional in reducing pressures, or stimulating personal learning and development (Llorens et al., 2006). Resources can be categorized into external ones (social and environmental support) and internal ones (coping strategies and self-management) and these two kinds of resources are positively associated with adaptation and personal wellbeing (Hsu and Tung, 2010). In accordance with JD-R theory, conservation of resources (COR) theory proposed that individuals tend to make efforts to maintain, preserve, and construct resources they value and there are many kinds of resources, including interpersonal resources such as supports from family and social relations and personal trait resources (Hobfoll, 2001). COR theory also suggests that individuals who lose resources are more likely to produce stress and result in burnout (Prapanjaroensin et al., 2017), and individuals with more resources tend to be involved in a positive loop—being less vulnerable to resource loss and more accessible to other resources. Bakker and Demerouti (2017) pointed out that job resources which include social support, opportunities for professional development, and performance feedback play a similar role as personal resources like self-efficacy and optimism. With regard to college context, social support could be attained as an external resource from a positive family environment or peer groups, while positive psychological capital (PsyCap), a kind of positive psychological trait/state manifested in individuals, which is composed of four aspects: hope, self-efficacy, resilience, and optimism, could be regarded as an important intrapersonal resource (Wu et al., 2021). A growing body of empirical evidence has also indicated that these resources are correlated and play as protective factors in coping with academic stressors and alleviating academic burnout (Cushman and West, 2006; He et al., 2018).

Peer support is generally defined by the fact that people with similar experiences can better relate and consequently offer assistance, practical advice and suggestions, and more authentic empathy and validation (Mead and MacNeil, 2006). For college students who leave home to lead an independent life in school communities, peers are an important source of support in various areas of functioning during college years and peer support is an essential interpersonal resource for individuals to rely on and trust (Lamis et al., 2016). As noted in social support theory, social support, as one of the effective ways to cope with life events that people perceive as threats, is a stable resource to decrease these threats and reduce strain and depression caused by them (Heaney and Israel, 2008). By helping build social and problem-solving skills from peers’ experiences, offering support and appraisal, and listening to intimate thoughts to promote connectivity (Kibret and Tareke, 2017), social support from peers protects students against mental health problems like depression, suicide, and anxiety (Roach, 2018). Peer support could enhance individual self-esteem and social competence, then reduce academic burnout (Kim et al., 2018). It was also shown that peer support partly predicts strong resilience in coping with academic stress and burnout (Lakey and Cohen, 2000; Heaney and Israel, 2008). Another essential benefit obtained from peer support is hope, a belief in a better future, which is created through contacting people who are recovering and having similar experiences, and who have found ways through their difficulties and challenges (Davidson et al., 2006). Besides, relationship patterns in families could be reflected among peers with similar connectedness, put it another way, students tend to follow the way family members get along in developing friendships and thus students living in families with low intimacy and adaptability are much more likely to live in a vicious cycle of isolation (Bell et al., 1988). Moreover, children’s positive experiences in an adaptive and cohesive family could enhance adjustment in peer relationships and better interact with optimal peer support to improve children’s well-being (Ye et al., 2021).

Positive psychological capital is a kind of personal trait resource involving four major components: hope, self-efficacy, optimism, and resilience (Luthans et al., 2007). The combination of four subcategories provides individuals with synergistic power, addressing two major questions: *Who are you*? (your actual self) and *Who do you want to be*? (your potential self) (Avey et al., 2010). Accordingly, individuals will be highly motivated to achieve one’s goal and actively cope with demands and pressures. Studies have demonstrated its benefits in individual behaviors, attitudes, academic achievement (Vanno et al., 2014), job performance (Luthans et al., 2010), and mental health (Newman et al., 2014). As stated in COR theory, people with enough personal psychological resources are not vulnerable to stress from resource loss (Halbesleben et al., 2014). For instance, self-efficacy, as one component of PsyCap, was found negatively correlated to academic burnout (Tuominen-Soini and Salmela-Aro, 2014). It could contribute to enhancing students’ confidence and ability to cope with stressful situations (Walburg, 2014), and low self-efficacy could lead to pessimistic thinking mode and coping strategies (Rahmati, 2015). Moreover, students with high resilience tend to opt for the deep approach and problem-focused strategies of learning which predict better school performance and less academic stress and burnout (de la Fuente et al., 2017). Additionally, people’s personal resources like self-efficacy, self-esteem, and optimism often travel in packs and emerge from nurturing social conditions like supportive families or peer groups and the loss and gain of resources both have a spiraling nature (Mishra, 2020). Family cohesion was found to affect individual resilience through perceived social support and self-esteem (Wei, 2021). Another study supported the argument that psychological resilience exerts a mediating effect on the link between family cohesion and adaptability and academic burnout with the cultivation of coping ability and emotional support (Hou and Liang, 2017). Besides, it was found in research among patients and relocating women that perceived social support was positively correlated to overall PsyCap and its components, hope, self-efficacy, resilience, and optimism (Foote et al., 1990; Schönfeld et al., 2017; Sagi et al., 2021). Furthermore, evidence from the Chinese college students’ sample showed that PsyCap significantly mediated the relationships between perceived social support and subjective wellbeing (Huang and Zhang, 2021). Peer support’s positive effect on enhancing self-efficacy and emotional adaptation was also evidenced (Wang, 2020).

**FIGURE 1:**
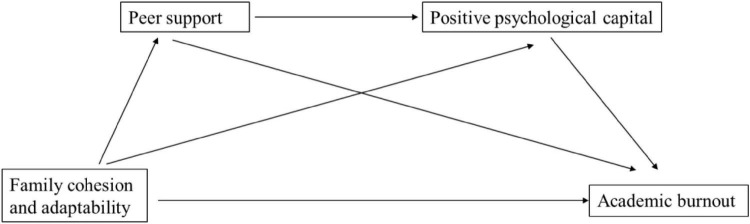
The proposed conceptual framework of family cohesion and adaptability, academic burnout, peer support, and positive psychological capital.

In summary, few research has explored the relationship between functional aspects of the family environment (i.e., family cohesion and adaptability) and academic burnout of college students, as well as the internal working processes or mechanism. Based on JD-R theory and COR theory, the present study aimed to examine the relationship between family cohesion and adaptability and academic burnout and to test the possible mediation roles of peer support (interpersonal resource) and PsyCap (intrapersonal resource) in their relationship. Specifically, we proposed the following hypotheses as depicted in Figure 1:

H1: Family cohesion and adaptability are negatively correlated to academic burnout.

H2: The negative relationship between family cohesion and adaptability and academic burnout was mediated by enhancing peer support.

H3: The negative relationship between family cohesion and adaptability and academic burnout was mediated by PsyCap.

H4: Peer support and PsyCap mediate the negative relationship between family cohesion and adaptability and academic burnout in serial. That is, students with a higher level of family cohesion and adaptability have more peer support, and then more PsyCap, which leads to less academic burnout.

## Materials and Methods

### Participants and Procedures

Participants were recruited from undergraduates at a university located in Wuhan city, central China. The research was carried out during the winter vacation of the 2019–2020 academic year when students were taking online courses because of the home quarantine of the coronavirus disease 2019 (COVID-19) epidemic. Researchers first obtained their teachers’ consent, then the announcement about an online questionnaire survey was delivered. Then, the trained psychological investigators recruited from their classes sent a survey link or QR code via online class groups. Students voluntarily participated in the study and signed the informed consent by clicking the “agree” button before starting the online survey. A total of 2,199 students completed the questionnaires. After deleting the cases who answered the questionnaire less than 240 s and failed the screening item (chose “no” on the item “I answered all the questions according to my situations”), a total of 1,971 valid cases (1,390 female and 581 male) with a mean age of 19.59 years old (*SD* = 1.13) were involved into data analysis. Table 1 presents the sample characteristics. Students of graduates (Year 4) did not involve in this study for most of the credits and courses have been finished and they were engaged in looking for jobs or preparing for postgraduate entrance exams or international study tests. This study adhered to the ethical principles of human subjects and was approved by the Ethics in Human Research Committee of Education and Counseling Center of Psychological Health, Zhongnan University of Economics and Law.

**TABLE 1:** Sample profile (*N* = *1971*).

Variable		Frequency	Percentage
Gender	Male	581	29.5
	Female	1390	70.5
Year of study	1	742	37.6
	2	724	36.7
	3	505	25.6
Major	Liberal arts and social sciences	1523	77.3
	Engineering and technology	448	22.7
Ethnics	Minority	211	10.7
	Han	1760	89.3
Religion	Yes	70	3.6
	No	1901	96.4
The only child	Yes	1051	53.3
	No	920	46.7
Family residential area	Municipalities/provincial capitals	547	27.8
	Prefecture-level city	516	26.2
	County-level cities	551	28
	Town	151	7.7
	Rural areas	206	10.5
Family monthly income (RMB, yuan)	2000 and below	75	3.8
	2001–4000	265	13.4
	4001–6000	296	15
	6001–8000	325	16.5
	8001–10000	336	17
	10001–20000	411	20.9
	20001 and above	263	13.3
Age (years)		Mean = 19.59, *SD* = 1.13, range (16–26)	

### Measures

#### Family Cohesion and Adaptability

Family cohesion and adaptability were measured using the Family Adaptability and Cohesion Evaluation Scales, Second edition Chinese Version (FACES II-CV) (Fei et al., 1991). The scale consists of 30 items (16 items for family cohesion and 14 items for family adaptability). Respondents rate each of the items about their family situations on a 5-point scale from 1 (“almost never”) to 5 (“almost always”). FACES II-CV was shown good psychometric properties with all the Cronbach’s α > 0.60. In this study, Cronbach’s α achieved 0.93 for the whole scale and 0.84 and 0.88 for the subscales of cohesion and adaptability, respectively.

#### Academic Burnout

Academic burnout was evaluated by Learning Burnout Scale (LBS), which was developed using a Chinese undergraduate sample (Lian et al., 2005) based on Maslach Burnout Inventory (Maslach et al., 1996). The scale consists of 20 items measuring emotional exhaustion (EE, i.e., feeling exhausted due to demands, 8 items), depersonalization (DP, i.e., having a cynical, detached attitude toward one’s study, 6 items), and reduced personal accomplishment (RPA, i.e., feeling incompetent or inefficacy as a student, 6 items). Respondents rate on a 5-point Likert scale ranging from 1 (“totally disagree”) to 5 (“totally agree”). In the current study, Cronbach’s α achieved 0.89 for the total scale, and 0.83, 0.75, and 0.73 for three subscales, respectively.

#### Positive Psychological Capital

Psychological capital was assessed by the Positive Psychological Capital Questionnaire (Zhang et al., 2010), a 26-item scale measuring four components: hope, optimism, self-efficacy, and resilience. Items were scored on a 7-point Likert scale ranging from 1 (“totally disagree”) to 7 (“totally agree”). Cronbach’s α achieved 0.92 for the total scale, and 0.77, 0.77, 0.85, and 0.83 for the subscales of self-efficacy, resilience, hope, and optimism, respectively. The total score was calculated by summing all the 26 items and a higher score represents more PsyCap.

#### Peer Support

Peer support was assessed as support from classmates, friends, and social groups, using a short 8-item scale modified from the widely used Chinese Social Support Scale (Xiao, 1994). Participants rate the degree to which each item is true of their peers on a 4-point Likert-type scale from 1 (“none”) to 4 (“much”). The scale was tested to be reliable and with good content validity and discriminant validity (Li et al., 2020). Cronbach’s α of this measure in this study was 0.79.

## Results

### Means, SDs, and Correlations Between Variables

The data analyses were conducted in SPSS Version 24.0. Means, SDs, and correlations between the main variables of interest were presented in Table 2. The surveyed college students reported moderate levels of academic burnout (total score = 54,83, EE = 22.18, DP = 17.09, and RPA = 15.56). As expected, family cohesion and adaptability were negatively related to academic burnout (*r* = −0.24, *p* < 0.01), and positively related to PsyCap (*r* = 0.41, *p* < 0.01) and peer support (*r* = 0.31, *p* < 0.01). PsyCap and peer support were negatively related to academic burnout (*r* = −0.65, *p* < 0.01 and *r* = −0.29, *p* < 0.01). PsyCap was positively associated with peer support (r = 0.46, *p* < 0.01). Thus, H1 was supported.

**TABLE 2:** Means, SDs, and correlations between variables.

	Variables	Mean	*SD*	1	2	3	4	5	6	7
1	Academic burnout	54.83	10.77	1	0.90**	0.87**	0.73**	−0.24**	−0.65**	−0.29**
2	EE	22.18	5.47		1	0.69**	0.46**	−0.11**	−0.49**	−0.23**
3	DP	17.09	3.87			1	0.52**	−0.21**	−0.54**	−0.25**
4	RPA	15.56	3.36				1	−0.35**	−0.66**	−0.28**
5	Family cohesion and adaptability	101.53	18.41					1	0.41**	0.31**
6	PsyCap	123.48	20.29						1	0.46**
7	Peer support	23.15	3.75							1

**p < 0.01.

### Hierarchical Regression Analyses

Hierarchical regression analyses were performed in SPSS to examine the relative contributions of demographic variables, family cohesion and adaptability, peer support, and PsyCap in the variances of academic burnout. Academic burnout was set as the dependent variable (DV), and all the independent variables (IV) were included in regression step by step. Four models were obtained. As shown in Table 3, in the first step, the demographic variables (i.e., gender, age, and year of study, etc.) were entered into the model, and only family monthly income was shown to be significant in explaining the variances of academic burnout (*b* = −0.524, *t* = −3.361, and *p* < 0.01). When family cohesion and adaptability was included in Step 2, the percentage of variance of academic burnout explained by the IVs were significantly increased from *R*^2^ = 0.008 in Model 1 to *R*^2^ = 0.063 in Model 2, family cohesion and adaptability was demonstrated to be a significant contributor (*b* = −0.141, *t* = −10.781, and *p* < 0.01). Then, after peer support was included as the IV in Step 3, the percentage of variance of academic burnout explained by the IVs were significantly increased in Model 3 (*R*^2^ = 0.114, ΔR^2^ = 0.109, ΔF = 112.481, and *p* < 0.01). Peer support was demonstrated to be significant in explaining the variance of academic burnout (*b* = −0.691, *t* = −10.606, and *p* < 0.01), while the significance of family monthly income was shown to disappear in Model 3, and the significant contribution of family cohesion and adaptability was decreased from *b* = −0.141 in Model 2 to *b* = −0.100 in Model 3. In last step, when PsyCap was included as the IV, it was shown to be significant in explaining the variance of academic burnout (*b* = −0.361, *t* = −33.216, and *p* < 0.01), while the significant contributions of family cohesion and adaptability and peer support were disappeared in Model 4. All the IVs of Model 4 together explained 43.4% of the variance of academic burnout (ΔR^2^ = 0.430, ΔF = 1103.272, and *p* < 0.01).

**TABLE 3:** Hierarchical regression analyses of academic burnout.

DV: Academic burnout												
IV	Model 1			Model 2			Model 3			Model 4		
	*b*	Beta	*T*	*B*	Beta	*t*	*b*	Beta	*t*	*b*	Beta	*t*
Constant	58.514		8.824**	72.639		11.045**	81.016		12.569**	100.198		19.312**
Gender	–0.206	–0.009	–0.380	–0.232	–0.010	–0.441	0.225	0.010	0.438	–1.867	–0.079	−4.496**
Age	0.093	0.010	0.300	0.194	0.020	0.642	0.224	0.024	0.765	0.051	0.005	0.219
Ethnics	0.060	0.002	0.075	0.047	0.001	0.060	0.337	0.010	0.443	0.425	0.012	0.698
Year of study	0.001	0.000	0.001	–0.106	–0.008	–0.247	–0.381	–0.028	–0.911	–0.547	–0.040	–1.637
Major	0.431	0.017	0.738	0.671	0.026	1.182	0.486	0.019	0.880	0.577	0.022	1.306
The only child	–0.346	–0.016	0.665	–0.889	–0.041	–1.749	–0.763	–0.035	–1.544	0.019	0.001	0.048
Religion	–1.513	–0.026	–1.142	–2.320	–0.040	–1.798	–1.690	–0.029	–1.345	–0.362	–0.006	–0.360
Family Residential area	0.024	0.003	0.108	–0.074	–0.009	–0.345	–0.027	–0.003	–0.130	–0.131	–0.015	–0.792
Family monthly income	–0.524	–0.085	−3.361**	–0.336	–0.054	−2.199*	–0.139	–0.022	–0.927	–0.106	–0.017	–0.888
Family cohesion and adaptability				–0.141	–0.242	−10.781**	–0.100	–0.170	−7.461**	0.021	0.036	1.847
Peer support							–0.691	–0.241	−10.606**	0.027	0.009	0.474
PsyCap										–0.361	–0.680	−33.216**
*R* ^2^		0.008		0.063				0.114			0.434	
ΔR^2^		0.003		0.059				0.109			0.430	
ΔF		1.744		116.238**			112.481**			1103.272**		

*p < 0.05,**p < 0.01; b, unstandardized coefficient; Beta, standardized coefficient.

### Mediation Analyses

Mediation effects were examined using Model 6 of SPSS macro PROCESS, a regression-based modeling tool with a bootstrap approach (Hayes, 2017). Academic burnout was set as the dependent variable with the functioning of the family environment as the independent variable, and peer support and PsyCap as two mediators, while family monthly income was controlled as a covariate for possible influence.

**FIGURE 2:**
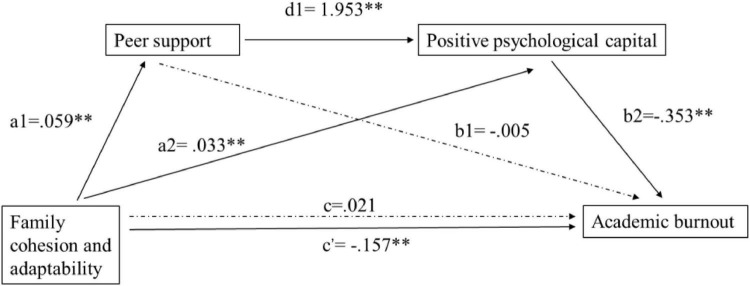
The serial mediation role of peer support and positive psychological capital in the relationship between family cohesion and adaptability and academic burnout and unstandardized beta values. c and c’ represent the direct effect and indirect effect of family cohesion and adaptability on academic burnout, respectively. ^**^p < 0.01.

As presented in Figure 2, the direct effect of family environment on academic burnout was not significant (c = 0.021, *p* = 0.06), but the indirect effect was significant (c’ = −0.157, *p* < 0.01). Family environment was positively related to PsyCap (a2 = 0.330, *p* < 0.01), and peer support (a1 = 0.059, *p* < 0.01), then led to more PsyCap (d1 = 1.953, *p* < 0.01), which finally contributed to lower academic burnout (b2 = −0.353, *p* < 0.01). However, the relationship between peer support and burnout was not significant (b1 = −0.005, *p* > 0.05). The results demonstrated that peer support alone did not mediate the effect of family environment on academic burnout with zero being among the 95% confidential interval (−0.008, 007). The total and specific indirect effects of family environment on academic burnout through three paths were presented in Table 4. Thus, H3 and H4 were supported, while H2 was not.

**TABLE 4:** Total, direct, and indirect effects of family cohesion and adaptability through peer support and positive psychological capital on academic burnout.

		Bootstrapping		
		95% confidential interval		
	Effect	Boot SE	Lower	Upper
Total effect	–0.136	0.013	–0.162	–0.111
Total direct effect	0.021	0.011	–0.001	0.043
Total indirect effect	–0.157	0.011	–0.179	–0.137
Family cohesion and adaptability→peer support→academic burnout	–0.000	0.004	–0.008	0.007
Family cohesion and adaptability →peer support→ PsyCap→academic burnout	–0.116	0.010	–0.136	–0.098
Family cohesion and adaptability →PsyCap →academic burnout	–0.041	0.005	–0.050	–0.032

## Discussion

In this study, we examined the association between the functioning of the family environment and academic burnout among college students and the underlying mediating mechanisms. Generally consistent with previous findings (Hou and Liang, 2017; Aunola et al., 2018), participants in families with higher cohesion and adaptability tend to show less emotional, behavioral, or cognitive burnout symptoms in learning. We further found that family cohesion and adaptability do not directly affect academic burnout, but indirectly exert effects through increasing intrapersonal resources (i.e., PsyCap) and interpersonal resources (i.e., peer support). Specifically, the proposed hypotheses H1, H3, and H4 were supported, that is, family cohesion and adaptability negatively relate to academic burnout, the relationship between family cohesion and adaptability and academic burnout was mediated by PsyCap, the relationship between family cohesion and adaptability and academic burnout was mediated by peer support and PsyCap in serial. H2 was rejected, that is, the relationship between family cohesion and adaptability and academic burnout was not mediated by peer support alone.

As expected, the current study confirmed the negative association between family cohesion and adaptability and academic burnout of college students. More importantly, it revealed two underlying mediating paths, one is through increasing PsyCap and the other one is through enhancing peer support and then increasing PsyCap. First, a family environment with high quality functioning (i.e., higher family cohesion and adaptability) leads to more PsyCap, which in turn alleviates academic burnout. Warmth, love, support, positive interactions, and communications in a functioning family can fulfill individuals’ innate psychological needs for connection, autonomy, and competence, which is crucial to achieve optimal psychological growth and health (Deci and Ryan, 2000). On the one hand, this kind of family environment will help shape positive self-concept and cultivate stable positive personal traits (such as hope, optimism, resilience, and self-efficacy) to deal with the demands and pressures in the process of growth and development and allow them to function positively. On the other hand, when students are suffering difficulties and adversities in college context, they, with the most reliable family connection and support network, can get timely comfort, encouragement, care, practical advice from family members, which would lead students to present a positive psychological state—PsyCap, that is, feeling confident in their ability (self-efficacy), believing that they will be able to tackle challenges at present or in the future (optimism), redirecting paths to facilitate goal achievement (hope), and bouncing back when faced with difficulties and adversities (resilience). Thus, PsyCap, a kind of intrapersonal psychological resource elicited in the family of higher cohesion and adaptability, in turn, leads students to experience lower academic burnout.

Moreover, the study indicated that a higher level of family cohesion and adaptability would increase peer support, which leads to more PsyCap and finally contributes to less academic burnout. According to COR theory, social support can both widen individuals’ pool of available resources and replace resources that have been lost or lacking (Alarcon et al., 2011). College life is often stressful and students would seek support from social networks to deal with the stressors, therefore peer support becomes a key interpersonal resource outside the family system. Family and peer systems were interconnected key factors in individuals’ developmental trajectories. Positive and functional family with cohesion and adaptability allows individuals to enjoy a warm and supportive family environment, prompting the psychological experience of productive and creative activities into a wider space, which results in a higher willingness to care for others and the community and an enhanced capability to establish and maintain positive relationships with peers (Lan et al., 2021). Healthy family functioning may help individuals develop trust in those who are perceived as supportive (Muscarà et al., 2018). College students from families with higher cohesion and adaptability were shown to have more peer support (Luo and Zhang, 2015).

Although myriads of studies have indicated social support’s significant effects on buffering stressors, some researchers have found that the direct effect on burnout symptoms in academic or job context was insignificant (Kim and Stoner, 2008; Alarcon et al., 2011; Pan and Zhang, 2021). This study also demonstrated that the negative relationship between family cohesion and adaptability and academic burnout was not mediated through peer support alone, but through the path that the effect of family functioning on peer support in increasing intrapersonal resource, that is, PsyCap, which in turn reduces academic burnout. Support, connection, sense of belongingness, and positive interaction in both interpersonal systems may help individuals fulfill psychological needs by obtaining positive emotional, cognitive, and instrumental resources in particular and thus have self-confidence, hope, optimism, and resilience to confront with demands and stressors that may lead to burnout. This result revealed the mechanism that both family functioning and peer support as the external resources could reduce depletion of energy and alleviate academic burnout through the internal psychological resource (i.e., PsyCap).

### Implications

Overall, this study extends research on the relationship between family functioning and academic burnout as well as the underlying mediating mechanism in college students. From the theoretical perspective, the findings supported that resources are important in coping with demands, stress, and reducing burnout and in particular extended our knowledge about how different kinds of resources (relational and personal) and sources of social support (support from a good family environment and peer group) interplay in reducing stress and academic burnout. Family environment with a high level of cohesion and adaptability, support from peers and interpersonal relationships, and PsyCap as individual personal trait resource can protect young adults against academic burnout and facilitate positive development. In practice, these findings also offer a new perspective on school educational and counseling services to reduce academic burnout. Even in college life students are spending an increasing amount of time with peers and building new friendships, family functioning remains as crucial as peer support in helping individuals cope with stress and academic burnout. Efforts should be made to encourage students to maintain frequent connections, active communications, and emotional bonding with their family members through well-developed internet tools and other effective ways, even if they live far away from home. For students with academic burnout or at risk, the school should contact their families and offer professional guidance on promoting parent–child interaction and communication. Campus should provide guidance to help students in developing healthy and harmonious relationships with roommates, classmates, and other peer groups, and various resources and opportunities should be available for students to discuss and communicate. It may also be helpful to develop training programs for peer-mentors and role models to help students adapt to study and life and then prevent and alleviate academic burnout. Moreover, since PsyCap is regarded as relatively malleable and open to development (Luthans et al., 2008), school counseling services should incorporate current positive psychological intervention programs with psychological capital interventions (or PCI). A cumulative body of research has evidenced that individuals’ PsyCap with components of hope, self-efficacy, optimism, and resilience can be enhanced through face-to-face or web-based PCI in samples of students and employees (Luthans et al., 2014; Dello Russo and Stoykova, 2015; Goertzen and Whitaker, 2015). Endeavors should be made in promoting individual positive perception, behavior, and emotions to mitigate the impact of stress, so as to prevent and reduce academic burnout.

### Limitations and Future Studies

However, certain limitations should be mentioned. First, cross-sectional data is difficult to determine the causal relationship, therefore further investigations are required to clearly understand the formation of academic burnout among college students. Second, the sample was drawn from a single university with certain characteristics, such as it being a highly competitive liberal arts and social science-oriented university and having more female students, which might be less generalizable to other universities and countries. Third, other important variables, such as the school-related or individual factors, were excluded from the current study and whether the COVID-19 pandemic and home quarantine would affect students’ family perception and academic burnout was unknown. Finally, the study was carried out in a special cultural setting—that is, China. Because parent–child relationships and friendships (Rubin et al., 2011) and personalities (such as the Five Big, extraversion, agreeableness, conscientiousness, neuroticism, and openness to experience) (Schmitt et al., 2007) are varied in different cultures, further cross-cultural studies are needed to add to our understanding of the development of academic burnout among college students.

## Conclusion

This research sought to find out the impact of family functioning (i.e., family cohesion and adaptability) on academic burnout in college students. The findings indicated a negative relationship between family cohesion and adaptability and academic burnout. Meanwhile, this relationship was mediated by PsyCap (intrapersonal resource) alone and by peer support (interpersonal resource) and PsyCap in serial. The current study provides a theoretical model for understanding how the family environment would affect academic burnout among young adults from the resource perspective. It also lends insights for school counseling services to promote youth mental health and positive development with regard to college students.

## Data Availability Statement

The original contributions presented in the study are included in the article/supplementary material, further inquiries can be directed to the corresponding author.

## Ethics Statement

The studies involving human participants were reviewed and approved by the Ethics in Human Research Committee of Education and Counseling Center of Psychological Health, Zhongnan University of Economics and Law (date of approval: December 06, 2019). The participants provided their written informed consent to participate in the study.

## Author Contributions

JY and XQT: conceptualization, investigation, and resources. JY, XQT, and XMT: methodology. XQT and JH: software. JY and YFW: validation. XQT, JH, and YFW: formal analysis. JY, YFW, and XQT: writing—original draft preparation. JY, YFW, YQW, and XQT: writing—review and editing. All authors have read and agreed to the published version of the manuscript.

## Conflict of Interest

The authors declare that the research was conducted in the absence of any commercial or financial relationships that could be construed as a potential conflict of interest.

## Publisher’s Note

All claims expressed in this article are solely those of the authors and do not necessarily represent those of their affiliated organizations, or those of the publisher, the editors and the reviewers. Any product that may be evaluated in this article, or claim that may be made by its manufacturer, is not guaranteed or endorsed by the publisher.
